# Concept, diagnosis and classification of bisphosphonate-associated osteonecrosis of the jaws. A review of the literature

**DOI:** 10.4317/medoral.21001

**Published:** 2016-01-31

**Authors:** Carmen Gavaldá, Jose V. Bagan

**Affiliations:** 1 Associate Professor of Oral Medicine. University of Valencia. Primary Care Dentist. Valencian Public Health Service. Valencia (Spain); 2 Chairman of Oral Medicine. University of Valencia. Head of the Department of Stomatology. Valencia University General Hospital. Valencia (Spain)

## Abstract

**Background:**

Bisphosphonates (BPs) and other antiresorptive agents such as denosumab are widely prescribed for the treatment of osteoporosis and are also used in patients with multiple myeloma and metastatic breast or prostate cancer for avoiding bone reabsorption and fractures that result in increased morbidity-mortality among such individuals.

**Material and Methods:**

We made a bibliographic search to analyze the concept, diagnosis and the different classifications for bisphosphonate-associated osteonecrosis of the jaws.

**Results:**

Osteonecrosis of the jaws (ONJ) is an important complication of exposure to BPs or other antiresorptive agents, and although its prevalence is low, it can pose management problems. The definition, diagnosis and classification of osteonecrosis have evolved since Marx reported the first cases in 2003.

**Conclusions:**

The present study offers a literature review and update on the existing diagnostic methods and classification of the disorder, with a view to facilitating earlier and more effective treatment.

**Key words:**Osteonecrosis, jaws, bisphosphonates.

## Introduction

Bisphosphonates (BPs) are stable pyrophosphate analogs that modulate bone metabolism, and are generally used to treat certain diseases involving bone reabsorption, such as osteoporosis or Paget’s disease (usually administered via the oral route), or hypercalcemia associated to different malignancies such as multiple myeloma and bone metastases secondary to solid tumors of the breast or other locations (via the intravenous route). Bisphosphonates fundamentally act by inhibiting bone reabsorption, with the limitation of osteoclast acti

vity, though they are also considered to exert an antiangiogenic effect (1,2). Other antiresorptive drugs apart from BPs are also used to treat osteoporosis, multiple myeloma and bone metastases. In this regard, denosumab (a RANKL inhibitor) has been included in the treatment guides as an option for preventing bone problems (e.g., hip or vertebral fractures), and is administered via the subcutaneous route once every 6 months for the ma-nagement of osteoporosis (1,3).

However, the use of both BPs and other antiresorptive drugs can produce adverse effects in the form of gastrointestinal disorders or osteonecrosis of the jaws (ONJ). The latter is defined as an area of exposed or necrotic bone that fails to heal within 8 weeks in patients who have received treatment with BPs in the absence of maxillary radiotherapy (4). The pathogenesis of ONJ remains unclear, though the suppression of osteoclast-mediated bone remodeling with consequent bone sclerosis and ischemia has been suggest as the likely causal mechanism. This moreover would explain the increased risk of ONJ when BPs are administered in combination with antiangiogenic agents such as bevacizumab or sunitinib (5).

- Frequency of appearance

BPs for the treatment of osteoporosis

The prevalence of ONJ is far greater in patients treated with intravenous BPs than in those who receive oral BPs; indeed, some authors consider the association between oral BPs and ONJ to be insignificant (6). As a result, the recommendations regarding dental treatment (e.g., surgery or dental implant placement) in such patients can be vague and lack firm supporting evidence (6).

BPs via the oral route: The prevalence of ONJ varies greatly (0.001-0.10%), depending on the literature source (7,8). With a treatment duration of four years or more, the reported prevalence is 0.21%, while the prevalence drops to 0.04% with shorter treatments (7). In a European multicenter study involving 470 cases of ONJ due to BPs, a total of 37 (7.8%) were attributed to oral BPs prescribed for the treatment of osteoporosis (6). The clinical significance of the oral route therefore should not be underestimated.

The incidence ranges from 1.04-69 cases per 100,000 patients/year (8). Kühl *et al.* (9) recorded a mean incidence of 0.12%, while other authors (10) have described incidences of between 0.0009%-0.034%.

Intravenous BPs for the treatment of osteoporosis: The ONJ prevalence in this case is greater, between 0-0.348% (8). The incidence ranges from 0-90 cases per 100,000 patients/year (8).

Intravenous BPs in cancer treatments

The prevalence of ONJ in cancer patients treated with intravenous BPs varies between 0.52-7.4%, depending on the source (3,5,11). The incidence in turn ranges from 0.8-12% (9,10).

On comparing denosumab with BPs in cancer patients, the former drug has been found to offer a greater decrease in bone events and superior safety in patients with kidney disease. However, the associated ONJ rate is similar to that observed with BPs, and hypocalcemia is comparatively more frequent (12,13).

The systemic risk factors for ONJ are the type of BP used, the administration route, the duration of treatment, the cumulative dose, the background disease for which the medication is prescribed, concomitant therapies (e.g., chemotherapy, corticosteroids, antiangiogenic agents, etc.), patient habits (smoking, alcohol, etc.), gender, age, genetic factors, and other disease conditions such as diabetes mellitus, rheumatoid arthritis, hemodialysis, etc. (5,14).

The local risk factors in turn include dentoalveolar surgery (especially extractions) – this being the leading risk factor for ONJ in cancer patients subjected to antiresorptive treatment – as well as dental and periodontal infection, and removable dentures. Anatomical factors (mandible, torus) also play a role (5,11,14).

The aim of this study is to offer an update on the concept, diagnosis and classification of ONJ due to BPs.

## Material and Methods

A literature search was made of the Medline-PubMed, Scopus and Cochrane databases, using the following key words: osteonecrosis, concept, diagnosis, classification, bisphosphonates, staging, jaws. The key words were validated in the MeSH and were combined using the boolean operators AND / OR.

The inclusion criteria were: articles published in English or Spanish in the period between 2006-2015, human studies and reviews, systematic reviews, metaanalyses and consensus documents. In addition, a manual search was made of the references cited in key articles, including relevant letters to the editor and case series. Articles centered on the etiopathogenesis and/or treatment of ONJ were not included.

**Figure 1:**
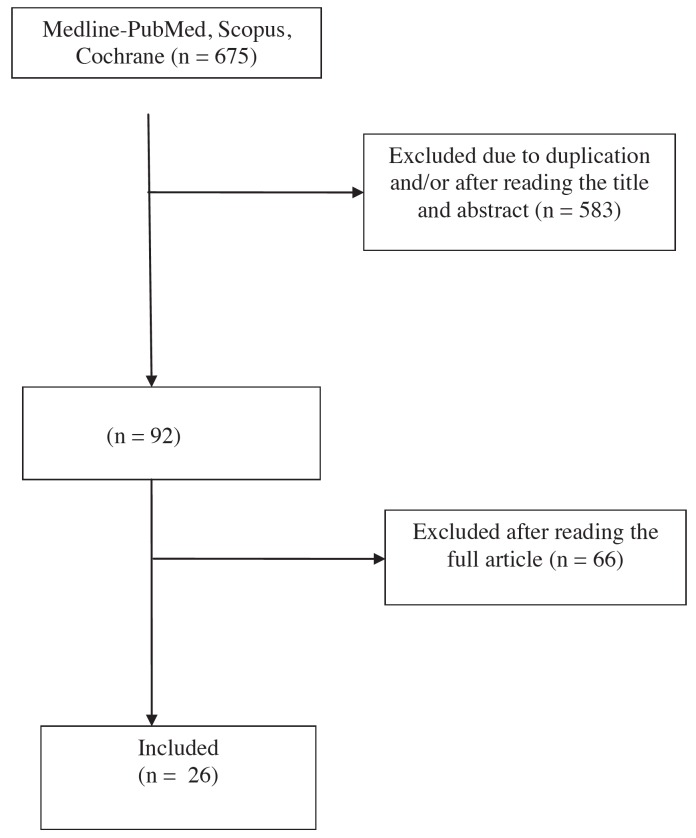
Article flowchart.

A total of 675 articles were identified. Of these, 583 were excluded due to duplication and/or after evaluation of the title and abstract. A full-text evaluation was made of the remaining 92 articles, with the final exclusion of 66 publications that were not considered to be sufficiently related to the aspects of ONJ which we aimed to analyze (Fig. 1).

## Results

Of the 26 articles selected for the present review, most were published in the year 2009 (n = 8, 30.8%). The authors were from the United States or Canada in 13 cases (50%), and from Europe (Spain, Italy, Denmark, United Kingdom) in 11 cases (42.3%). One publication was from Japan and one was an international consensus document (United States, Canada, Germany, New Zealand, Japan, Italy, United Kingdom, Jordan, Austria, Denmark, Finland, Switzerland and Saudi Arabia). Ten articles addressed all three aspects reviewed in our study (concept, diagnosis and treatment) (38.5%), while the rest addressed one or more of them. Of the total articles selected, 8 were consensus documents, four were letters to the editor, four were case series, one was a case-control study, 5 were reviews, two were clinical cases, and two were retrospective studies ( Table 1).

**Table 1:**
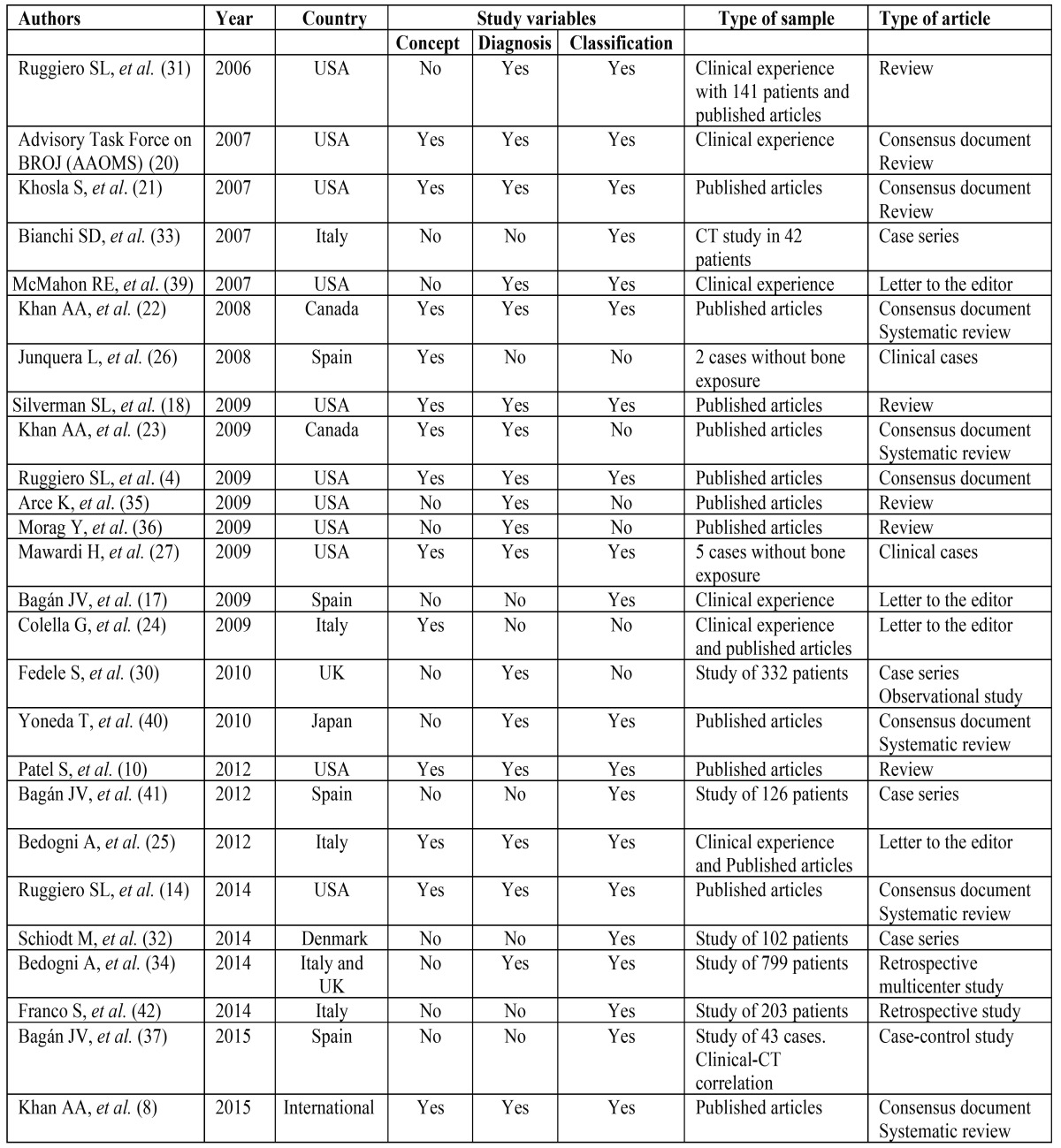
Articles selected for the review and their characteristics.

## Discussion

- Concept and diagnosis

A number of terms have been used in reference to osteonecrosis of the jaws due to bisphosphonate exposure, including bisphosphonate-associated osteonecrosis of the jaws (BAONJ), bisphosphonate-related osteonecrosis of the jaws (BRONJ), bisphosphonate-induced osteonecrosis of the jaws (BIONJ), bisphosphonate-rela-ted osteonecrosis (BRON), or simply bisphosphonate osteonecrosis (BON) (15).

The first cases of ONJ due to BPs were published by Marx in 2003 (16), and since then there has been a growing number of articles on this subject (17). Since the year 2006 different societies and expert panels have proposed a number of clinical descriptions for defining this new disease entity (18). As an example, in 2006 the American Dental Association (ADA) considered the typical clinical presentation of ONJ to include pain, swelling of the soft tissues, infection, tooth mobility, suppuration and bone exposure. Likewise in 2006, the Australia and New Zealand ONJ work group (19) underscored the lack of a clear definition of the disease. The authors suggested the definition of ONJ as an “area of exposed bone persisting for over 6 weeks”. The condition was to be suspected in patients with bone exposure in the maxillofacial region following oral surgery. Other symptoms such as pain and infection could also be present.

In 2006 the American College of Rheumatology (ACR) reported that ONJ typically manifests as an intraoral lesion with the exposure of white-yellowish bone, sometimes associated to the presence of an intra- or extra oral fistula. Likewise in 2006, the American Association of Endodontists (AAE) indicated that patients with ONJ present at least one of the following characteristics: ulceration of the mucosa with bone exposure in the upper maxilla or mandible, pain or swelling, infection and suppuration or sensory alterations (18). It is thus clear that no agreement has been reached regarding the definition of the disease. In the year 2007 the American Association of Oral and Maxillofacial Surgeons (AAOMS), in its position document on BRONJ, defined the latter as the exposure of necrotic bone in the maxillofacial region persisting for more than 8 weeks, in patients with current or past bisphosphonate therapy and no antecedents of radiotherapy of the maxillary region (20). That same year, the American Society for Bone and Mineral Research (ASBMR) defined a “confirmed case” of BRONJ as the presence of an area of exposed bone in the maxillofacial region failing to heal within 8 weeks after having been identified by a health professional in a patient with current or past treatment with BPs and no antecedents of radiotherapy of the maxillary region. A “suspected case” in turn was defined as an area of exposed bone in the maxillofacial region, present for less than 8 weeks, and identified by a health professional in a patient with the same characteristics as described above (21).

In 2008 the Canadian Association of Oral and Maxillofacial Surgeons published a consensus document with management guidelines referred to BRONJ in which the “confirmed case” and “suspected case” definitions introduced by the ASBMR were maintained (22). In 2009 this same work group published a review on the subject in which the same definitions were maintained without changes (23).

The AAOMS, likewise in 2009, published an update on the subject without modifying the definition which they had proposed two years earlier, and which has been maintained up until 2014 (4). However, the AAOMS did introduce a new stage (referred to as stage 0), corresponding to patients with symptoms but no exposed bone. Authors such as Colella *et al.* in 2009 and Bedogni *et al.* in 2012, among others, considered that the term BRONJ should be redefined in order to include patients in stage 0 (24,25). Based on clinical cases published by other authors and on the habitual clinical findings, they suggested that the definition of BRONJ should include not only cases with exposed bone but also those with necrotic bone in which bone exposure has not yet occurred (24). Furthermore, they considered that the diagnosis and classification should be based not only on the clinical picture but also on the radiological findings (24,25).

Other investigators such as Bagán *et al.* in 2009, Junquera and Gallego in 2008, and Mawardi *et al.* in 2009 (17,26,27), also suggested that ONJ may manifest in the absence of bone exposure, particularly in the early stages, with fistulas, pain and radiographic alterations.

On the other hand, cases have since been published in which ONJ has been associated to other antiresorptive agents such as denosumab or cancer drugs with antiangiogenic effects such as sunitinib, sorafenib, bevacizumab or sirolimus. As a result, some authors have proposed other terms such as drug-induced osteonecrosis of the jaws or osteonecrosis of the jaws associated to antiresorptive agents, in reference to this disorder (10,15).

In 2014, the AAOMS proposed a change in nomenclature in favor of the term medication-related osteonecrosis of the jaws (MRONJ) (14).

In addition to incorporating this change in name of the disease, the AAOMS update of 2014 modified its definition. In this regard, a patient is considered to have MRONJ if all of the following conditions are met (14):

- Current or past treatment with antiresorptive or antiangiogenic drugs.

- Exposed bone or intra- or extraoral fistulization in the maxillofacial region communicating with the bone and persisting for more than 8 weeks.

- No history of maxillary radiotherapy or clear maxillary metastatic disease.

However, in 2015 the International Task Force on Osteonecrosis of the Jaw defined ONJ as follows (8):

- Exposed bone in the maxillofacial region that fails to heal in 8 weeks after identification by a health professional.

- Exposure to an antiresorptive agent.

- No history of craniofacial radiotherapy.

The diagnosis is essentially clinical (28). On the other hand, it must be taken into account that there may be one or more sites of bone exposure (29). Furthermore, these sites may remain asymptomatic for prolonged periods of time (weeks, months or even years), or clinical signs and symptoms may manifest before clinically detectable ONJ develops. Such signs and symptoms consist of pain, bone and/or gingival swelling, erythema, suppuration, soft tissue ulceration, intra- or extraoral fistular trajectories, tooth mobility, paresthesia and even anesthesia, in the absence of any apparent dental/periodontal cause.

The radiographic findings range from variable radiotransparency or radioopacity to the absence of radiological signs. In the absence of bone exposure, these findings alone were not regarded as sufficient to diagnose BRONJ (21).

At present, the latest update of the AAOMS corresponding to 2014 (14) considers that the presence of these manifestations, even in the absence of bone exposure (equivalent to stage 0 of the 2009 classification) is indicative of prodromal BRONJ, and that over time up to 50% of these patients will progress towards disease stages 1, 2 or 3.

Clinically, the exposure of necrotic bone mostly occurs after dentoalveolar surgery (extractions or the placement of dental implants), though it can also be spontaneous. The most frequent location is the mandible (62-82% of the cases), maxilla (8-18%), or both (up to 20% of the cases), with a predominance of the molar and premolar regions. The exposed bone is generally colonized by oral bacteria, giving rise to secondary infections (11,28).

The differential diagnosis of ONJ must be made with other conditions such as alveolar osteitis, sinusitis, osteomyelitis, periodontitis/gingivitis, periapical disease caused by caries, mucositis, osteoradionecrosis, temporomandibular joint disease and certain forms of cement-bone dysplasia with secondary sequestration phenomena (8,21). Accordingly, the patient case history and the clinical examination remain the most sensitive tools for diagnosing ONJ (8,30).

The two most controversial aspects in the diagnosis of ONJ since the publication of the first case series have been: 1) the diagnosis of ONJ in the absence of bone exposure; and 2) the need for radiological or imaging confirmation of the diagnosis. These two aspects will be examined in greater detail below.

- Diagnosis of ONJ in the absence of exposed bone:

Most authors have accepted the definition of ONJ proposed by the AAOMS in 2007, i.e., an area of exposed bone in the maxillofacial region that fails to heal within 6-8 weeks, in a patient with current or past treatment with BPs but without head and neck radiotherapy. However, as has been commented above, some investigators have suggested that ONJ may manifest without bone exposure, particularly in the early stages of the disease. As an example, in 2008 Junquera and Gallego (26) described two patients with bone sequestration that could be clinically and radiographically classified as corresponding to stage 3 ONJ, but without bone exposure. In these cases pain and swelling were the main symptoms. The authors suggested that there is a variant of ONJ without bone exposure. Based on their clinical experience, Bagán *et al.* in 2009 (17) proposed that the three stages of the 2006 classification of Ruggiero (31) should include patients with intraoral fistulas but no bone exposure, since these patients otherwise could not be assigned to any stage.

In 2009, Mawardi *et al.* (27) described 5 patients subjected to treatment with BPs who developed deep periodontal pockets, tooth mobility or intraoral fistulas with or without suppuration, with swelling in some cases, and with radiographic alterations (sequestration, sclerosis, lack of post-extraction socket healing), but without exposure of necrotic bone. After several months bone exposure occurred in the same zone. The authors regarded these cases as corresponding to early stage BAONJ and proposed modifying the definition of BONJ to include a new category: “suspected BONJ” or stage 0s, since in the same way as Bagán *et al.* (17), they were unable to assign the patients to any of the three established ONJ stages.

On the other hand, Fedele *et al.* in 2010 (30) studied 332 patients with ONJ and found up to 28.9% of the subjects to have clinical manifestations consistent with the purported ONJ variant without bone exposure. The clinical manifestations in decreasing order of frequency were maxillary pain, fistulization, bone expansion and gingival swelling. In addition, the symptoms developed spontaneously without previous extractions or surgery, and in 29.1% of the cases no radiological alterations were observed in the panoramic X-ray or computed tomography explorations. Manifest bone exposure was seen over time (up to two years of follow-up) in 53.1% of these patients. According to the authors, these findings may have a significant impact upon the existing epidemiological data and on the design of future studies.

According to Patel *et al.* (10), the absence of exposed bone in patients with ONJ can produce a delay in diagnosis, prolong the disease and cause it to become refractory to treatment. They proposed a diagnostic and therapeutic approach to cases of ONJ without bone exposure based on the symptoms, assessment of the risk factors, the radiographic evidence, and patient refractoriness to medical treatment. The authors suggested a modification of the AAOMS staging or classification system, as will be seen below. In 2014, Schiodt *et al.* (32) indicated that the proportion of ONJ without bone exposure may be high (29-45% of all cases of ONJ), and that this fact could result in potential under reporting of the disease in epidemiological studies. The authors evaluated 102 patients with ONJ, with and without bone exposure, and established comparisons between the two groups in order to determine whether they corresponded to the same disease condition or not. No significant differences were found between the two groups in terms of the demographic data, symptoms, clinical and radiological characteristics, histopathological findings or survival. As a result, they concluded that both presentations form part of the same disease and proposed a new ONJ classification including patients without bone exposure, as will be commented below.

Lastly, as we have seen, the AAOMS update of the year 2014 (14) modifies the definition of ONJ, with the inclusion of cases of ONJ without bone exposure, though the classification does not contemplate such presentations.

- Need for radiological or imaging confirmation of the diagnosis:

In 2006, Ruggiero *et al.* (31), in an article presenting guidelines for the diagnosis, staging and treatment of ONJ, described the existence of both early and late radiographic maxillary changes that could simulate other disorders (periapical disease, osteomyelitis, myeloma or metastatic disease). In the case of important bone involvement, regions with a mottled appearance (similar to the pattern seen in osteomyelitis) could be found. Likewise, widening of the periodontal ligament and bone osteosclerosis could be observed, particularly in the region of the hard lamina. However, according to these authors, the radiographic changes were not evident until important bone alteration had developed. They consequently suggested that the panoramic and periapical X-ray studies might not reveal significant changes in the early stages of ONJ. According to Khosla *et al.* (21), in the presence of well established disease, imaging techniques are not needed for diagnostic purposes since the presence of exposed bone and other clinical signs and symptoms suffice to identify ONJ. Nevertheless, they recognized that such techniques may be of importance in the early identification of ONJ.

Other authors have used imaging techniques in patients of this kind, including computed tomography (CT), magnetic resonance imaging (MRI), scintigraphy and panoramic and periapical X-rays. As an example, Bianchi *et al.* in 2007 (33) studied 32 patients with ONJ, comparing the alterations found in panoramic X-rays versus CT. The latter technique was found to be far superior, with the detection of lesions in almost twice as many patients. In all cases CT detected structural alterations of the trabecular bone and cortical erosion. In comparison, the panoramic X-rays failed to detect bone sequestration in almost half of the cases. Intense periosteal reaction was a common finding, and oroantral communications could also be observed. In 2012, Bedogni *et al.* (25) proposed a new classification of ONJ, as will be seen below, based particularly on the CT findings. Likewise, in 2014 Bedogni *et al.* (34) conducted a large retrospective multicenter study of the CT findings in 799 patients with ONJ. They found that the severity (extension) of the lesions can be identified and measured with CT much more accurately than with panoramic X-rays or clinical inspection, as proposed by some classifications – including that of the AAOMS of 2009. The authors attempted to correlate the findings with the stages proposed by the AAOMS in 2009, and concluded that these stages are unable to correctly identify disease extent or involvement, except in stage 3.

On the other hand, in 2009 Arce *et al.* (35) conducted a review of the findings in ONJ with different imaging techniques. According to these authors, the radiographic findings are not specific. Intraoral and panoramic X-rays may show widening of the lamina dura and of the periodontal ligament, osteolysis, diffuse sclerosis and a lack of post-extraction socket healing. The more advanced the disease the greater the sclerosis and widening of the mandibular canal. Zones of ONJ can be identified in quadrants without exposed bone. Computed tomography offers a three-dimensional view of the extent of the lesion and can detect minor sequestrations. Focal sclerosis with a disorganized trabecular pattern is present in the early stages of the disease, and neck adenopathies and masticatory muscle thickening simulating a tumor mass can be detected. Magnetic resonance imaging in turn can detect bone marrow and soft tissue involvement, nerve bundles and adenopathies. Bone scintigraphy with technetium-99 shows enhanced radio nuclide uptake between 10-14 days before bone mineral loss becomes significant enough to be detectable on X-rays. The problem with scintigraphy is its lack of specificity and low resolution (35,36). In 2015, Bagán *et al.* (37) analyzed the degree of sclerosis in different ONJ stages using CT, and investigated the relationship between the degree of sclerosis, the clinical symptoms and the extent of the radiotransparencies in 43 cases - establishing comparisons with a group of 40 controls without bone lesions. The patients with ONJ had more intense sclerosis than the controls (*p*<0.01). Furthermore, the degree of sclerosis increased with the clinical stage of ONJ and was correlated to the extent of the radiotransparency.

Morphological analysis of the necrotic bone (sequestrations) using micro-CT has been unable to demonstrate the existence of unique distinguishing features in patients with ONJ in different stages (38).

- Classification

In 2006, Ruggiero *et al.* proposed an ONJ classification comprising three stages (31): stage 1 = bone exposure but without signs or symptoms of infection; stage 2 = bone exposure/necrosis with clinical evidence of infection; stage 3 = the above manifestations and also alterations such as pathological fractures, extraoral fistulas or osteolysis extending to the inferior mandibular margin.

In 2007 the AAOMS adopted this classification (20), though in addition to the group of patients with BRONJ (with its three stages), it included another group of patients comprising individuals at risk. These patients were defined as subjects without evident exposed or necrotic bone or symptoms, but who have been treated with oral or intravenous BPs.

In the year 2009, the AAOMS added a stage 0 to its classification, involving alterations (pain, tooth mobility, fistulas, radiographic changes, etc.) that may have been due to treatment with BPs, but without exposed bone. The risk of progression towards more advanced stages of the disease was not known at that time (4).

Other classifications have also introduced the idea that ONJ may be present despite the absence of bone exposure. As an example, McMahon *et al.* considered that an early stage of ONJ with or without symptoms may exist in which bone exposure has not yet occurred, since the first bone changes are found at marrow level, not in cortical bone, and that early detection of this stage could improve the patient prognosis and treatment (39). They also considered that imaging techniques and histological studies are needed to more precisely categorize the different ONJ stages. The authors proposed 6 stages ( Table 2), but also considered that a stage 0 could be useful for identifying patients at risk. Mawardi *et al.* suggested the inclusion of a stage 0s as corresponding to “suspected BRONJ”. This stage in turn would comprise two subcategories: 0ss in the presence of symptoms, and 0sa in the absence of symptoms (27). Bagán *et al.* in turn included fistular lesions in stages 1, 2 and 3, though without bone exposure, and subdivided stage 2 into stages 2a and 2b according to whether the condition responded to conservative management or not (17). Yoneda *et al. *accepted the definition of ONJ of the AAOMS, but proposed the introduction of four stages in accordance with the situation of the disease in Japan at that time (40). This classification is basically the same as that of the AAOMS of 2009, but stage 0 moreover includes hypoesthesia or anesthesia of the lower lip as symptom and the presence of deep periodontal pockets as clinical sign ( Table 2). Other authors such as Bagán *et al.* in 2012 aimed to validate the classification of the AAOMS of 2009 with a retrospective study of 126 cases of ONJ due to intravenous and oral BPs, comparing both groups and determining whether all the cases could be assigned to one or other of the proposed stages (41). More cases of ONJ without exposed bone were observed in the oral bisphosphonate group, with a larger number of advanced cases (stages 2 or 3) in the intravenous oral bisphosphonate group. In addition, 6 cases could not be assigned to any of the stages, for despite the presence of extraoral fistulas and mandibular fracture, no exposed bone was identified. The authors consequently proposed a new modification of the classification of Ruggiero *et al.* (4), with the inclusion in stage 3 of the term “exposed and necrotic bone or oral fistula without exposed bone” ( Table 2). Other classification proposals are described below:

**Table 2:**
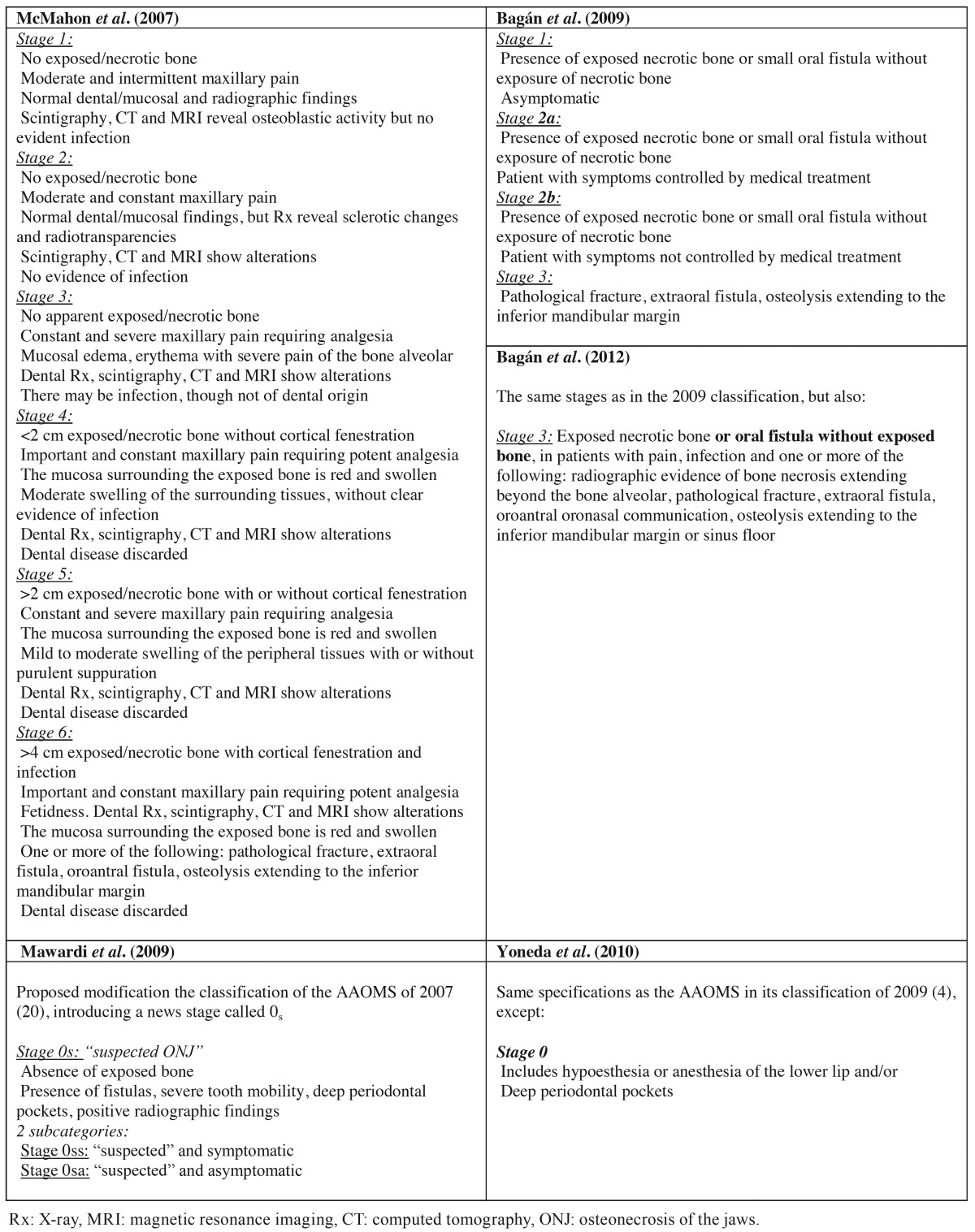
Proposals for modification of the ONJ classification of the AAOMS (4,20) by McMahon *et al.* (39), Bagán *et al.* (17,41), Mawardi *et al.* (27) and Yoneda *et al.* (40).

- Bedogni *et al.* in 2012 proposed a new classification with three stages (25): stage 1 = focal ONJ, stage 2 = diffuse ONJ, and 3 = complicated ONJ. In addition to clinical findings, this classification includes CT imaging findings and eliminates stage 0. According to these authors, the clinical manifestations of pain and suppuration should not be used to differentiate between stages, since they only define symptomatic or asymptomatic forms of BRONJ within one same stage. This contri-butes to avoid patient migration from stage 1 to stage 2 or vice versa (ping-pong effect). These authors fundamentally used the CT findings to classify the patients. The presence of bone sequestration was not regarded as a sign of complex BRONJ ( Table 3).

**Table 3:**
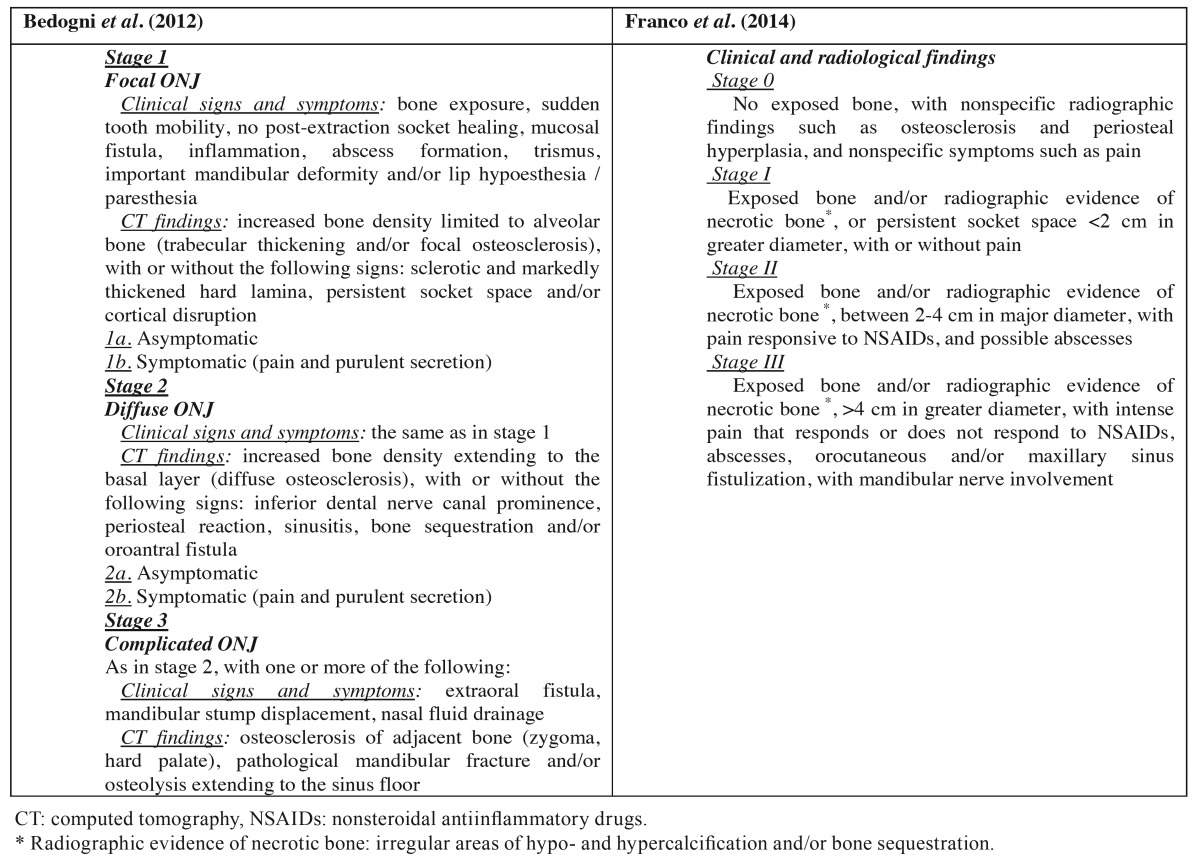
Proposals for the classification of ONJ according to Bedogni *et al.* (25) and Franco *et al.* (42).

- According to Franco *et al.* in 2014, most of the existing classifications are useful from the clinical and diagnostic perspective, but none of them offer a surgical orientation for the treating surgeon (42). They proposed a new dimensional staging system, classifying the lesions by size following panoramic X-ray and CT evaluation, with a view to making treatment decisions easier ( Table 3).

- In 2012, Patel *et al.* modified the classification of the AAOMS of 2009 with the purpose of incorporating patients without bone exposure and of guiding treatment (10). They distinguished between patients with and without bone exposure, and in the latter group those individuals without symptoms were classified as corresponding to stage 1, while those with symptoms were assigned to stages 2 or 3 ( Table 4).

**Table 4:**
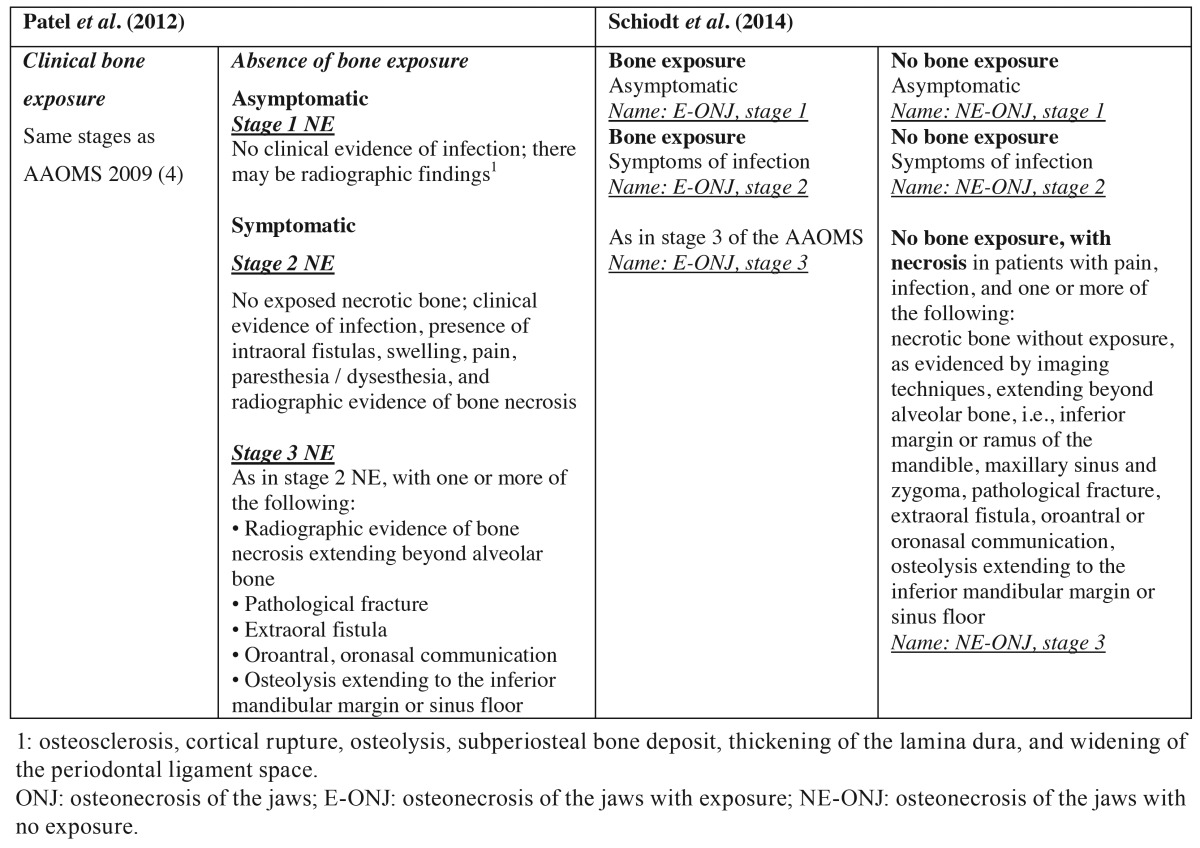
Proposals for the staging of ONJ according to Patel *et al. *(10) and Schiodt *et al.* (32).

- As has been commented, Schiodt *et al.* in 2014 considered ONJ with and without bone exposure to correspond to one same disease entity (32). Accordingly, they modified the classification of Patel *et al.* (10), eliminating stage 0 and classifying patients both with and without bone exposure in stages 1, 2 or 3 ( Table 4).

The classification or staging system currently proposed by the AAOMS (14), and which is correlated to therapeutic strategies specific of each stage, is as follows:

- At risk: Patients subjected to antiresorptive or antiangiogenic treatment via the oral or intravenous route, and with no symptoms or apparent bone necrosis.

- Stage 0 (disease variant without bone exposure): No clinical evidence of necrotic bone, though with clinical findings, radiographic changes and nonspecific symptoms.

Among the symptoms:

- Tooth pain in the absence of a dental cause.

- Maxillary bone pain that may irradiate to the region of the temporomandibular joint.

- Pain of the maxillary sinuses that may be associated to inflammation and thickening of the sinus walls.

- Altered neurosensory function.

Among the clinical findings: 

- Tooth mobility that cannot be explained by periodontitis.

- Periapical or periodontal fistulas not associated to pulp necrosis secondary to trauma, caries or restorations.

Among the radiographic findings:

- Loss or reabsorption of alveolar bone that cannot be explained by periodontitis.

- Changes in trabecular-dense bone pattern, with no formation of new bone in extraction sockets.

- Zones of osteosclerosis in alveolar bone or around the basal layer.

- Thickening or opacification of the periodontal ligament (thickening of the lamina dura, sclerosis, and reduction of the periodontal ligament space).

- Stage 1: Exposed bone or intra- or extraoral fistulization in the maxillofacial region penetrating to the bone, in asymptomatic patients without evidence of infection. In addition, radiographic findings such as those described in stage 0 may be observed in alveolar bone.

- Stage 2: Exposed bone or intra- or extraoral fistulization in the maxillofacial region penetrating to the bone, with infection evidenced by pain and erythema in the region or exposed bone with suppuration. In addition, radiographic findings such as those described in stage 0 may be observed in alveolar bone.

- Stage 3: Exposed bone or intra- or extraoral fistulization in the maxillofacial region penetrating to the bone, with pain, infection and at least one of the following signs:

- Necrotic bone extending beyond the alveolar bone (inferior margin or ramus of the mandible, maxillary sinus and zygoma)

- Pathological fracture

- Extraoral fistula

- Oroantral or oronasal communication

- Osteolysis extending to the inferior margin of the mandible or sinus floor

As can be seen, no unified classification or staging system has yet been established for use by all professionals – though most studies are based on the classification of the AAOMS. In coincidence with other authors such as Bedogni *et al.* (25), Patel *et al.* (10) and Schiodt *et al.* (32), we consider that stage 0 should be suppressed and that ONJ should be classified into three stages regardless of whether there is bone exposure or not. Furthermore, it would be advisable to establish the diagnosis not only on the basis of the clinical data but also on the findings of the CT scan, since the latter technique offers greater information on the extent and severity of the disorder.

Further studies and consensus are therefore needed with a view to adopting a single international classification allowing the conduction and comparison of epidemiological studies, and contributing to the treatment decision making process.
